# *Pseudomonas aeruginosa* biofilm-deficient mutants undergo parallel evolution during chronic infection

**DOI:** 10.1128/jb.00520-25

**Published:** 2026-05-28

**Authors:** Erin S. Gloag, Christopher W. Marshall, Nanami Kubota, Stacie E. Deaver, Brennan Deshotel, Vaughn S. Cooper, Daniel J. Wozniak

**Affiliations:** 1 Department of Biomedical Sciences and Pathobiology, Virginia-Maryland College of Veterinary Medicine, Virginia Tech 1757, Blacksburg, Virginia, USA; 2 Department of Biological Sciences, Marquette University 5505, Milwaukee, Wisconsin, USA; 3 Department of Microbiology and Molecular Genetics, University of Pittsburgh School of Medicine 12317 , Pittsburgh, Pennsylvania, USA; 4 Center for Evolutionary Biology and Medicine, University of Pittsburgh School of Medicine, Pittsburgh, Pennsylvania, USA; 5 Department of Microbial Infection and Immunity, The Ohio State University 2647, Columbus, Ohio, USA; 6 Department of Microbiology, The Ohio State University 2647, Columbus, Ohio, USA; Dartmouth College Geisel School of Medicine, Hanover, New Hampshire, USA

**Keywords:** type IV pili, lipopolysaccharide, adaptation, biofilm, filamentous phage

## Abstract

*Pseudomonas aeruginosa* readily adapts to infection by acquiring stable and heritable mutations. Previously, we discovered that the first adaptations in a porcine wound model were rugose small-colony variants (RSCVs) caused by mutations in the *wsp* operon. These mutants overproduce Pel and Psl biofilm exopolysaccharides that improve defense against host responses. To identify other mechanisms of host adaptation that lead to hyperbiofilm phenotypes, we created a mutant with an activated *wsp* pathway but unable to produce these exopolysaccharides (PAO1Δ*wspF*Δ*pelA*Δ*pslBCD*). Porcine wounds were infected with this mutant, and biopsies were sampled at days 7, 14, and 35. Colony variants were isolated from the wound, peaking at approximately 5% of the total *P. aeruginosa* population, and whole genome sequencing revealed that these variants had acquired mutations in genes in lipopolysaccharide and type IV pili biosynthesis, with *wzy* and *pilU* genes being most commonly targeted. PAO1 *pilU* mutants were associated with a hyperbiofilm phenotype that outcompeted the parental strain, and PAO1 *wzy* mutants were associated with a hyperbiofilm phenotype and increased tolerance to host antimicrobial products. We further identified that several variants had acquired large genome deletions that spanned up to 320 consecutive genes and other variants with high copy numbers of Pf6 filamentous phage. Together our results suggest that the hyperbiofilm phenotype is adaptive in chronic infections and that *P. aeruginosa* has redundant and diverse pathways to generate this phenotype.

**IMPORTANCE:**

We demonstrate that in a porcine full-thickness thermal injury wound model, a *Pseudomonas aeruginosa* mutant deficient in biofilm formation undergoes adaptive evolution by acquiring mutations that alter the outer membrane, either type IV pili (T4P) or lipopolysaccharide (LPS) mutations, that restores the deficient biofilm phenotype. We also observe a striking degree of mutational parallelism, at both the biosynthetic pathway and gene level, indicating the strong selective pressures experienced by these pathways during chronic wound infection.

## INTRODUCTION

*Pseudomonas aeruginosa* is an opportunistic pathogen, and evidence suggests that infections are established in compromised individuals through environmental reservoirs of the organism (1). Upon colonizing a host, *P. aeruginosa* often diversifies, acquiring beneficial adaptations that promote survival and persistence. An example of this is *P. aeruginosa* evolution and adaptation in response to colonizing the cystic fibrosis (CF) lung (2, 3). In the CF lung, the general paradigm is that *P. aeruginosa* evolves to become less virulent to promote chronic persistence (4). In line with this, specific genes or pathways are repeatedly targeted, with mutations commonly identified in genes associated with antibiotic resistance, virulence and secreted factors, quorum sensing, and of particular relevance to this study, motility and surface attachment and cell wall or lipopolysaccharide synthesis (2, 3), suggesting that mutations in these genes are under selection during infection.

Furthermore, mutations leading to increased biofilm and a hyperbiofilm phenotype are enriched in the CF lung (2, 3). In *P. aeruginosa*, one of the mediators controlling biofilm formation is the small signaling molecule c-di-GMP. The general paradigm is that low levels of c-di-GMP promote a planktonic lifestyle, while high levels of c-di-GMP promote a biofilm lifestyle (5). High c-di-GMP levels have pleotropic signaling affects (5), however specific to *P. aeruginosa* and biofilm formation, high c-di-GMP results in increased production of Pel and/or Psl polysaccharides and adhesin proteins, such as CdrA, subsequently promoting increased biofilm formation (6, 7). Furthermore, mutations leading to continued high c-di-GMP levels result in a rugose small-colony variant (RSCV) phenotype (Fig. S1). RSCVs are a distinct category of small-colony variants (SCVs). Multiple mutations have been associated with the small-colony phenotype, including metabolism and those that affect growth kinetics. However, all RSCVs that have been identified to-date have acquired mutations leading to elevated c-di-GMP (6, 8–12). Furthermore, due to the sustained high c-di-GMP levels, RSCVs overproduce both Pel and Psl, and subsequently are associated with a hyperbiofilm phenotype (13) and increased tolerance to antimicrobials (14). In contrast, mutations resulting in a small-colony phenotype do not always result in hyperbiofilm formation (15). In the CF lung, *P. aeruginosa* RSCVs of are frequently isolated that have mutations in the *wsp* system (2, 3). The *wsp* system is a chemosensory system that senses membrane stress and regulates c-di-GMP production upon surface attachment (6, 16). Mutations in *wspF, wspA,* and *wspE* that result in continued activation of WspR, the diguanylate cyclase of the Wsp system, and subsequently the RSCV phenotype, are routinely identified in *P. aeruginosa* CF clinical isolates, indicating that these mutations result in adaptive phenotypes, and that the Wsp pathway experiences strong selective pressure during infection (2, 3). Furthermore, *P. aeruginosa* RSCVs have been isolated from a range of infections other than CF pulmonary infections, including ventilator-associated pneumonia (17, 18), urinary tract infections (18, 19), catheter-associated urinary tract infections (20, 21), and canine otitis media (22). Hyperbiofilm variants, consistent with RSCVs, have also been isolated from osteomyelitis (23). Therefore, since RSCVs are considered to be adaptations to the biofilm lifestyle (13, 15), forming recalcitrant biofilms appears to be a common adaptation to chronic infections.

In our prior work, we used a porcine full-thickness thermal injury model to identify beneficial mutations that promote *P. aeruginosa* persistence during chronic infection (24, 25). Using variant colony morphology as an indicator for adaptive variation, we identified RSCVs as the only variant colony phenotype to emerge across the 28-day infection (24). Whole genome sequencing revealed that mutations in the *wsp* chemosensory system, leading to the RSCV phenotype, were the first mutations to be selected in the infection (24). As the overproduction of Pel and Psl by RSCVs results in hyperbiofilm formation (26), in the current study, we determined the genetic targets of *P. aeruginosa* adaptation to the wound in the absence of Pel and Psl exopolysaccharides to test alternative pathways of hyperbiofilm formation.

## RESULTS

### Exopolysaccharide-independent small colony variants (SCVs) are selected during porcine chronic wound infections

**Fig 1:**
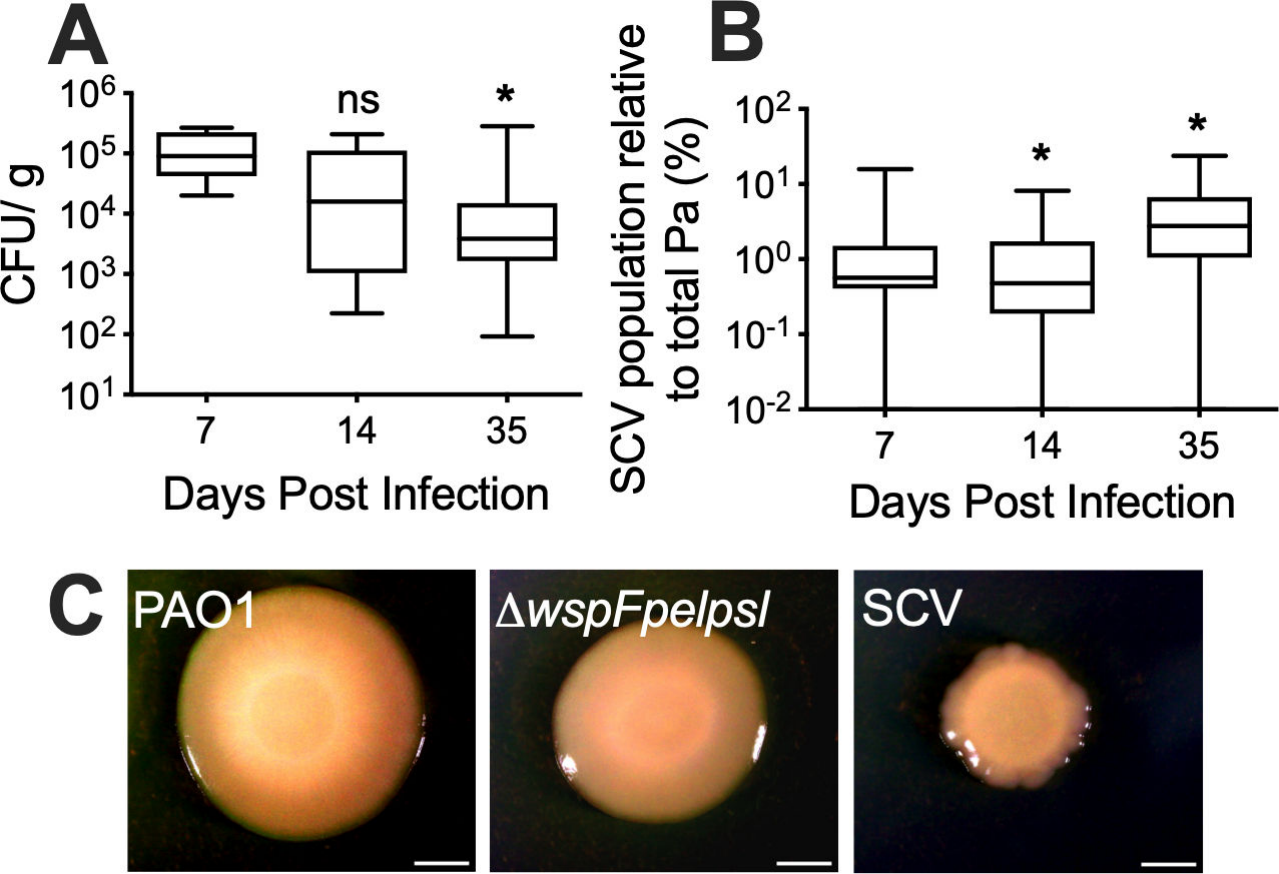
MPAO1Δ*wspFpelpsl* burden in a chronic wound infection. Porcine wounds were inoculated with MPAO1Δ*wspF*Δ*pelA*Δ*pslBCD*, and biopsies were sampled from the wounds at 7, 14, and 35 days post-infection. (**A**) Biopsies were homogenized and *P. aeruginosa* burden was enumerated CFU/gram tissue. (**B**) SCV subpopulation screened from homogenized tissue at each time point, expressed as a percentage of the total *P. aeruginosa* population. Three biopsies were sampled from a total of four wounds per time point. *N* = 12, per time point. Significance was determined using a one-way ANOVA with a Tukey’s multiple comparison post-hoc test. *P-value < 0.05, ns indicates no significance. Comparisons are to 7 dpi. (**C**) Representative colony morphologies of SCVs isolated from porcine wounds compared with wild-type PAO1 and Δ*wspFpelpsl* ancestor (labeled). Representative small-colony variant is SCV-71. Scale bar indicates 2 mm.

We previously determined that mutations in the *wsp* operon, resulting in the RSCV phenotype, were among the earliest mutations to be selected during chronic infection (24). We hypothesized that the fitness of these variants is associated with the hyperbiofilm phenotype resulting from the overproduction of Psl and Pel exopolysaccharides. We therefore wanted to identify other pathways leading to hyperbiofilm phenotypes that experience selection during infection. To achieve this, we assessed how a *wsp* mutant, with increased c-di-GMP but unable to produce Psl and Pel exopolysaccharides, adapted to the infection environment. Using a porcine thermal injury chronic wound model (27), wounds were inoculated with MPAO1Δ*wspF*Δ*pelA*Δ*pslBCD* (Δ*wspFpelpsl*), and the infection burden monitored 7, 14, and 35 days post-infection (dpi). Due to the absence of Pel and Psl polysaccharides, this mutant is deficient in biofilm formation, despite high intracellular c-di-GMP levels (12). This deficiency in biofilm formation resulted in an approximately 2-log reduction in bacterial burden in the wound (Fig. 1A) compared with previous infections in this model using wild-type *P. aeruginosa* (28). However, despite the lower bacterial burden, wounds still remained chronically colonized with Δ*wspFpelpsl* for the duration of the infection, with approximately 10^4^ CFU/g tissue recovered 35 dpi (Fig. 1A).

To determine if populations founded by Δ*wspFpelpsl* adapted to the wound environment, variant colony morphology was used as an indicator for evolved variants that emerged during the infection. Homogenized biopsy samples were grown on *Pseudomonas* isolation agar and screened for colony variants. Small-colony variants (SCVs) were the only variant morphology observed (Fig. 1B through C). SCVs were isolated across all three time points, and the phenotype was stable across three passages on non-selective growth media, suggesting that the SCV phenotype was due to heritable mutation(s) (Fig. 1B). Across the analyzed time points, the SCV frequency was the highest at 35 dpi, at approximately 5% of the total *P. aeruginosa* population (Fig. 1B). To determine if the SCV subpopulation experienced positive selection in the wound, selective coefficients were determined as a measure of relative fitness (equation 1) using a range of potential starting SCV frequencies (Table 1) (24). This revealed that across all three time points, SCVs likely had a selection rate >0.1 (i.e., 10% fitness advantage per generation) for all potential frequencies, indicating that SCVs experience strong positive selection in the wound.

**TABLE 1:** Selection rate (r) of SCVs from porcine wounds, inferred from potential starting frequencies

Starting frequency (SCV: ancestor)	r (mean ± SD) days post-infection		
	7	14	35
1:10^5^	0.96 ± 0.15	0.50 ± 0.14	0.26 ± 0.05
1:10^6^	1.28 ± 0.16	0.65 ± 0.10	0.31 ± 0.03
1:10^7^	1.60 ± 0.19	0.81 ± 0.09	0.36 ± 0.03
1:10^8^	1.92 ± 0.23	0.96 ± 0.09	0.41 ± 0.05

### Exopolysaccharide-independent SCVs acquire mutations in LPS O-antigen and type IV pili

**Fig 2:**
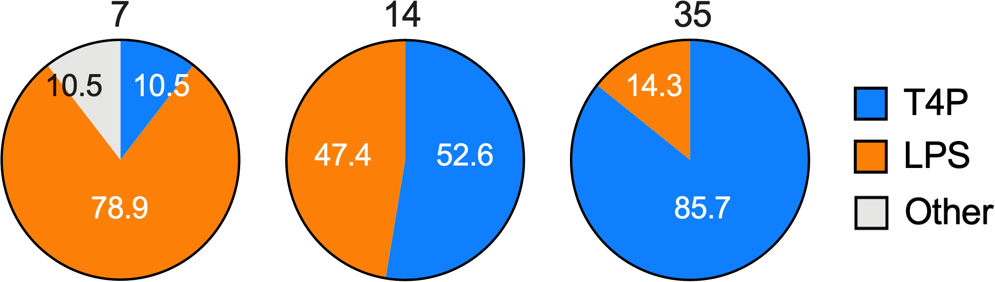
Classification of putative SCV mutations. Thirty-nine representative SCVs were sequenced. Mutations predicted to be responsible for variant colony morphologies could be categorized as either mutations in the T4P (blue) or LPS (orange) biosynthetic pathways, or other (gray). The number of mutations in either pathway is expressed as a percentage (labeled) of the total identified SCV mutations at either 7, 14, or 35 dpi (labeled). A total of 14, 15, and 10 isolates were sequenced from each time point, respectively. Individual mutations are listed in Table 2.

**Fig 3:**
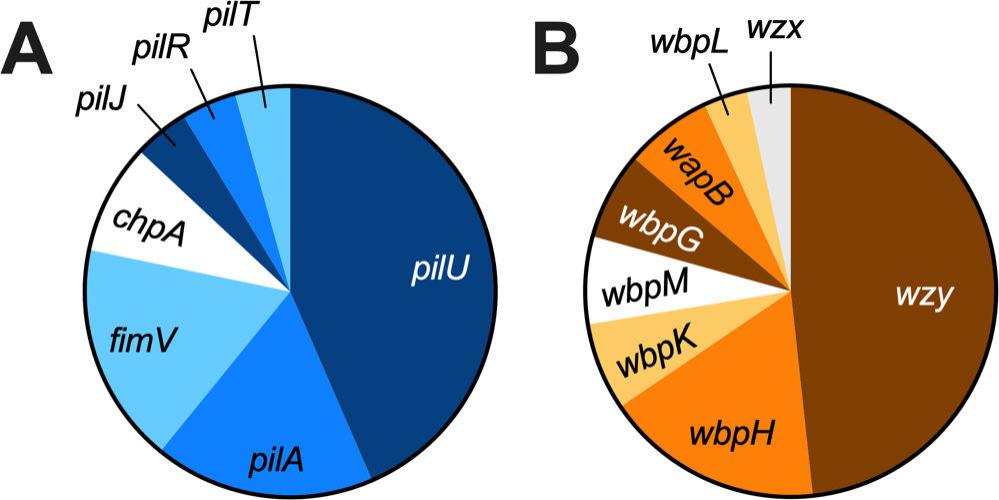
Frequency of genes with nonsynonymous SCV mutations. Frequency of driver mutations in genes (labeled) in either the (**A**) T4P or (**B**) LPS biosynthetic pathways, expressed as a percentage of the total number of mutations identified across the 39 sequenced SCVs, combined across all three time points. Individual mutations are listed in Table 2. Percentage of each gene is as follows: (**A**) *pilU* 43.5; *pilA* 17.4; *fimV* 17.4; *chpA* 8.7; *pilJ* 4.3; *pilR* 4.3; *pilT* 4.3; (**B**) *wzy* 48.3; *wbpH* 17.2; *wbpK* 6.9; *wbpM* 6.9; *wbpG* 6.9; *wapB* 6.9; *wbpL* 3.4; *wzx* 3.4. In total, 24 isolates had mutations in T4P genes, and 26 isolates had mutations in LPS genes.

**Fig 4:**
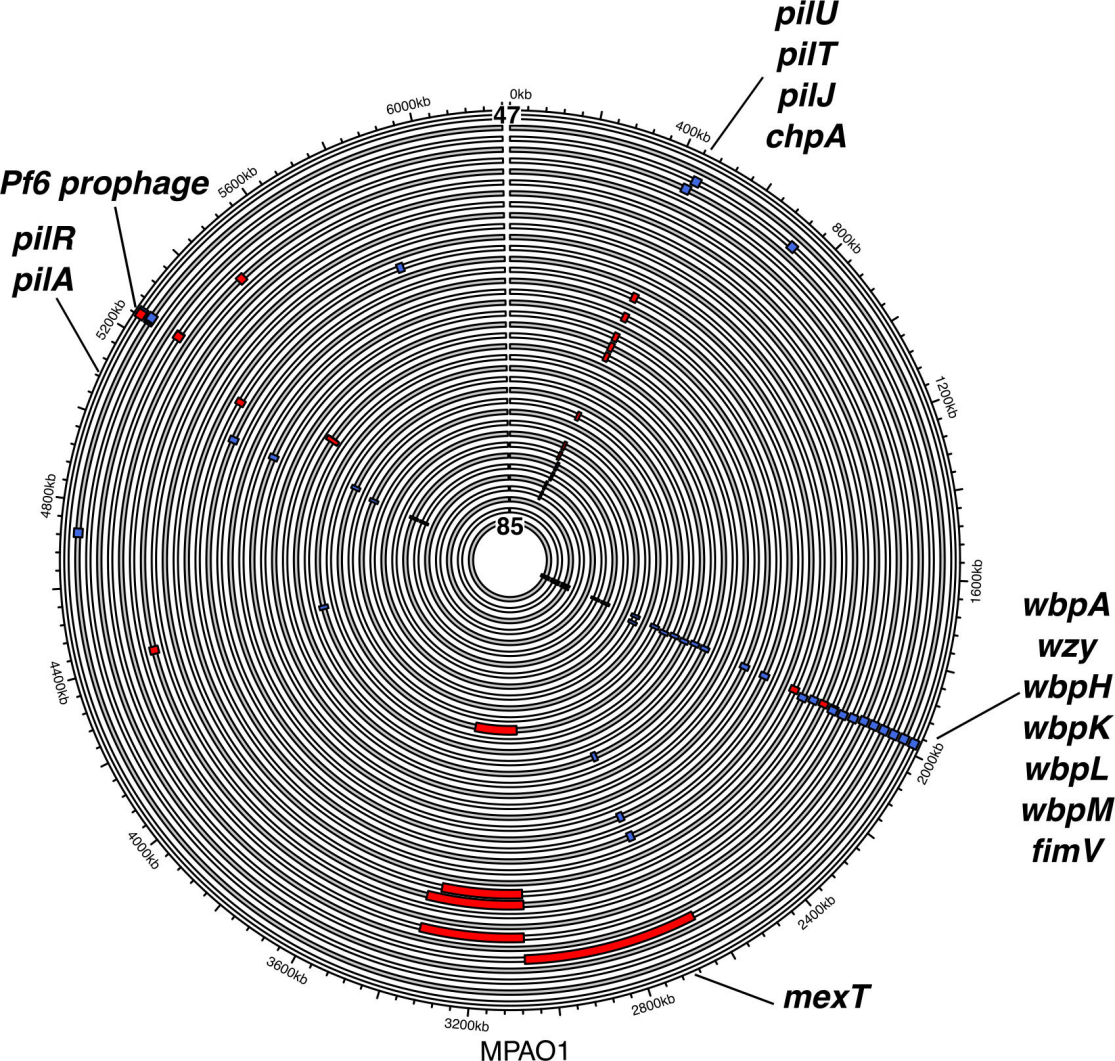
Parallel evolution across MPAO1Δ*wspFpelpsl* isolates. Circular genome map of each sequenced SCV, represented by a double black line, aligned to the MPAO1 genome. For simplicity, every second SCV is labeled. Mutations listed in Table 2 are represented; red are deletions; blue are small insertions or SNPs. Labeled are the locations of specific genes. Large deletions for SCVs 52, 54, 57, 58, and 73 begin at bp locations 2,637,522; 3,072,354; 3,069,871; 3,072,771; and 3,069,919 on the MPAO1 genome, respectively.

To determine the mutation(s) responsible for the SCV phenotype, whole genome sequencing was performed on 39 randomly selected SCVs (Table 2). This revealed that most putative driver mutations responsible for the SCV phenotype occurred in genes involved in either the type IV pili (T4P) or lipopolysaccharide (LPS) biosynthesis pathways (Fig. 2). Furthermore, mutations in genes in the T4P pathway became more prevalent at the later time points (Fig. 2). Specifically, *pilU* (Fig. 3A) and *wzy* (Fig. 3B) were the most frequently mutated genes belonging to the T4P and LPS pathways, respectively. PilU is an ATPase that powers retraction of the pilus (29), while Wzy is the polymerase for the synthesis of LPS O-antigen (30). The mutational parallelism in these two biosynthetic operons (Fig. 4), observed across *P. aeruginosa* isolates, wounds, and time points, is a strong indicator that T4P and LPS are under selection in this strain in the chronic wound environment.

**TABLE 2:** Mutations identified in SCVs

Sample			Driver mutation(s)				Secondary mutations			
Day	SCV	Wound*^a^	Locus	Gene	Annotation	Mutation	Locus	Gene	Annotation	Mutation
7	47	1;1	MPAO1_RS09245	wzy	O-antigen polymerase	(C)6→7	MPAO1_RS24190		Pf replication initiator	Δ379 bp
							MPAO1_RS24230/ MPAO1_RS24235		Hypothetical protein/protein phosphatase 2C domain containing protein	(GAAGCCAGTCGAAACTTGG)1→2
	48	1;2	MPAO1_RS09245	wzy	O-antigen polymerase	(A)7→8	MPAO1_RS21845 / MPAO1_RS21850		Phage destabilizing protein/DUF5447 family protein	G→A
							MPAO1_RS24190 / MPAO1_RS24195		Hypothetical protein/zonular occludens toxin family protein	A→G
							MPAO1_RS24220 / MPAO1_RS24225		Hypothetical protein/DUF5447 family protein	G→C
							MPAO1_RS24230 / MPAO1_RS24235		Hypothetical protein/protein phosphatase 2C domain‑containing protein	(GAAGCCAGTCGAAACTTGG)1→2
							MPAO1_RS24240		Serine/threonine‑protein kinase	(C)7→8
	49	1;3	MPAO1_RS03100	agtC	ABC transporter permease	IS110-like element insertion				
	50	1;3	MPAO1_RS09245	wzy	O-antigen polymerase	(C)6→7		23S rRNA		+AAAACGTTGGACGCATTAACAA TAAAAAAAAAA
			MPAO1_RS02105	pilJ	Chemotaxis chemoreceptor	A551V (GCG→ GTG)				
	51	2;1	MPAO1_RS02030	pilU	Type IV pilus ATPase	R176C (CGC→TGC)				
			MPAO1_RS09245	wzy	O-antigen polymerase	(C)6→7				
	52	2;1	MPAO1_RS09245	wzy	O-antigen polymerase	(A)7→8	MPAO1_RS24240		Serine/threonine‑protein kinase	(C)6→7
							MPAO1_RS12465 - MPAO1_RS14095			Δ434,361 bp
							MPAO1_RS28990		Lambda repressor	Δ168 bp
	53	2;3	MPAO1_RS09245	wzy	O-antigen polymerase	(C)6→7				
			MPAO1_RS25225	retS	Hybrid sensor histidine kinase/response regulator	Δ87 bp				
	54	3;1	MPAO1_RS09270	wbpH	Glycosyltransferase	(T)5→4	MPAO1_RS14095 - MPAO1_RS15330			Δ273,282 bp
	55	3;2	MPAO1_RS09285	wbpK	Probable NAD-dependent epimerase/dehydratase	L208Q (CTG→CAG)				
			MPAO1_RS20320	wapB	Glucosyltransferase	Δ53 bp				
	56	4;1	MPAO1_RS09270	wbpH	Glycosyltransferase	Δ1 bp				
			MPAO1_RS09290	wbpL	Glycosyltransferase	(G)9→10				
	57	4;1	MPAO1_RS09250- MPAO1_RS09270	wzx - wbpH		Δ3,332 bp	MPAO1_RS14090 - MPAO1_RS15340			Δ278,477 bp
	58	4;1	MPAO1_RS09270	wbpH	Glycosyltransferase	W300* (TGG→TGA)	MPAO1_RS14100 - MPAO1_RS15175			Δ 237,893bp
	59	4;2	MPAO1_RS09300	wbpM	Polysaccharide biosynthesis protein	(GCTTGCG) 1→2				
	60	4;3	MPAO1_RS09245	wzy	O-antigen polymerase	Δ261 bp	MPAO1_RS26980	amgS	Two-component system sensor histidine kinase	R182L (CGC→CTC)
							MPAO1_RS23920	cdrA	Two-partner secretion system adhesin	Δ8 bp
14	61	1;2	MPAO1_RS23380	pilA	Type 4a pilus biogenesis protein	S64G (T→C)	MPAO1_RS12725	oprN	Multidrug efflux RND transporter outer membrane subunit	(CGGGCGAAGGCCACGCGCAGG)1→2
	62	1;3	MPAO1_RS02030	pilU	Type IV pilus ATPase	Δ11 bp				
	63	1;3	MPAO1_RS09245	wzy	O-antigen polymerase	(C)6→7	MPAO1_RS12740	mexT	Multidrug efflux system transcriptional regulator	A179T (GCC→ACC)
	64	1;3	MPAO1_RS02030	pilU	Type IV pilus ATPase	Δ11 bp				
	65	1;3	MPAO1_RS09245	wzy	O-antigen polymerase	(A)7→8				
			MPAO1_RS23380	pilA	Type 4a pilus biogenesis protein	S64G (AGC→GGC)				
	66	1;3	MPAO1_RS02030	pilU	Type IV pilus ATPase	Δ11 bp				
	67	2;1	MPAO1_RS02030	pilU	Type IV pilus ATPase	Δ11 bp				
	68	2;1	MPAO1_RS02030	pilU	Type IV pilus ATPase	Δ11 bp				
	69	2;2	MPAO1_RS09245	wzy	O-antigen polymerase	(C)6→7	MPAO1_RS12740	mexT	Multidrug efflux system transcriptional regulator	P127L (CCG→CTG)
							MPAO1_RS28990		Lambda repressor	Δ238 bp
							MPAO1_RS24240		Serine/threonine‑protein kinase	(C)7→8
	70	2;2	MPAO1_RS09245	wzy	O-antigen polymerase	(A)7→6	MPAO1_RS28990		Lambda repressor	Δ35 bp
	71	3;2	MPAO1_RS09285	wbpK	Probable NAD-dependent epimerase/dehydratase	L208Q (CTG→CAG)				
			MPAO1_RS20320	wapB	Glucosyltransferase	P119L (CCC→CTC)				
	72	3;3	MPAO1_RS09270	wbpH	Glycosyltransferase	T152M (ACG→ATG)				
	73	4;3	MPAO1_RS09300	wbpM	Polysaccharide biosynthesis protein	+G	MPAO1_RS14095 - MPAO1_RS15330			Δ240,207 bp
			MPAO1_RS23510	pilR	Two-component system response regulator	T431M (ACG→ATG)				
	74	4;3	MPAO1_RS09265	wbpG	N-acetyl sugar amidotransferase	T177A (ACT→GCT)				
			MPAO1_RS02025	pilT	Type IV pilus twitching motility protein	Δ8 bp				
	75	4;3	MPAO1_RS23380	pilA	Type 4a pilus.biogenesis protein	S64G (AGC→GGC)				
35	76	1;2	MPAO1_RS09455	fimV	Type IV pilus assembly protein	+G	MPAO1_RS09220	wbpA	UDP-N-acetyl-D-glucosamine 6-dehydrogenase	M1M (ATG→ATT)
	77	2;1	MPAO1_RS02030	pilU	Type IV pilus ATPase	Δ11 bp				
	78	2;1	MPAO1_RS02030	pilU	Type IV pilus ATPase	Δ11 bp				
	79	2;1	MPAO1_RS09245	wzy	O-antigen polymerase	(C)6→7				
			MPAO1_RS02115	chpA	Chemotaxis signal transduction system protein	G1231C (GGC→TGC)				
			MPAO1_RS23380	pilA	Type 4a pilus biogenesis protein	S64G (AGC→GGC)				
	80	2;1	MPAO1_RS09245	wzy	O-antigen polymerase	(A)7→8				
			MPAO1_RS02115	chpA	Chemotaxis signal transduction system protein	Δ36 bp				
			MPAO1_RS23380	pilA	Type 4a pilus biogenesis protein	S64G (AGC→GGC)				
	81	3;1	MPAO1_RS02030	pilU	Type IV pilus ATPase	Δ11 bp				
	82	3;1	MPAO1_RS02030	pilU	Type IV pilus ATPase	Δ11 bp				
	83	4;1	MPAO1_RS09455	fimV	Type IV pilus assembly protein	+C	MPAO1_RS09220	wbpA	UDP-N-acetyl-D-glucosamine 6-dehydrogenase	M1M (ATG→ATT)
	84	4;3	MPAO1_RS09455	fimV	Type IV pilus assembly protein	+C	MPAO1_RS09220	wbpA	UDP-N-acetyl-D-glucosamine 6-dehydrogenase	M1M (ATG→ATT)
	85	4;3	MPAO1_RS09455	fimV	Type IV pilus assembly protein	+C	MPAO1_RS09220	wbpA	UDP-N-acetyl-D-glucosamine 6-dehydrogenase	M1M (ATG→ATT)

^*a*^ Numbers refer to wound and biopsy. For example, 1;1 indicates wound 1, biopsy 1. For each time point 4 wounds were sampled, wounds 1 and 2 are from animal 1 and wounds 3 and 4 from animal 2. For each wound 3 biopsies were sampled.

Sequencing of the SCVs revealed that many single isolates had acquired multiple mutations (Table 2). To determine the phenotypes and fitness effects associated with *pilU* and *wzy* mutations, and the contribution of the Δ*wspFpelpsl* background, the most common mutation in each gene was recreated in MPAO1 (referred subsequently here as PAO1), Δ*pelA*Δ*pslBCD* (Δ*pelpsl*), Δ*wspF,* and Δ*wspFpelpsl* backgrounds. The most common mutation in *pilU* was an 11 bp deletion of bases 560–570 (*pilU*_Δ560-570_) (Table 2). In the PAO1 and Δ*wspFpelpsl* backgrounds, the *pilU*_Δ560-570_ mutant had increased levels of surface T4P, indicative of hyperpiliation, compared with the parent (Fig. S2A). Introduction of wild-type *pilU in trans* restored piliation levels to that of the parent, indicating that *pilU*_Δ560-570_ is a loss-of-function mutation (Fig. S2A), consistent with previous observations of *pilU* mutants (31, 32). Similarly, in both the PAO1 and ΔwspFpelpsl backgrounds, the *pilU*_Δ560-570_ mutant had a SCV morphology, which could be complemented by introduction of wild-type *pilU in trans* (Fig. S2B). Together, this indicates that *pilU*_Δ560-570_ is a loss-of-function mutation that is responsible for the SCV morphology (Fig. S2A and B). The most common mutation in *wzy* was a single bp insertion at position 620 (*wzy*_620insC_) (Table 2). This resulted in a loss of O-antigen, or B-band LPS, production (Fig. S3A). Introduction of wild-type *wzy in trans* restored O-antigen production to that of PAO1 (Fig. S3A). In both the PAO1 andΔ*wspFpelpsl* backgrounds, the *wzy_620insC_* mutation resulted in an SCV morphology, which could be complemented by introduction of wild-type *wzy in trans* (Fig. S3B). Together, this indicates that *wzy*_620insC_ is a loss-of-function mutation that is responsible for the SCV phenotype.

**Fig 5:**
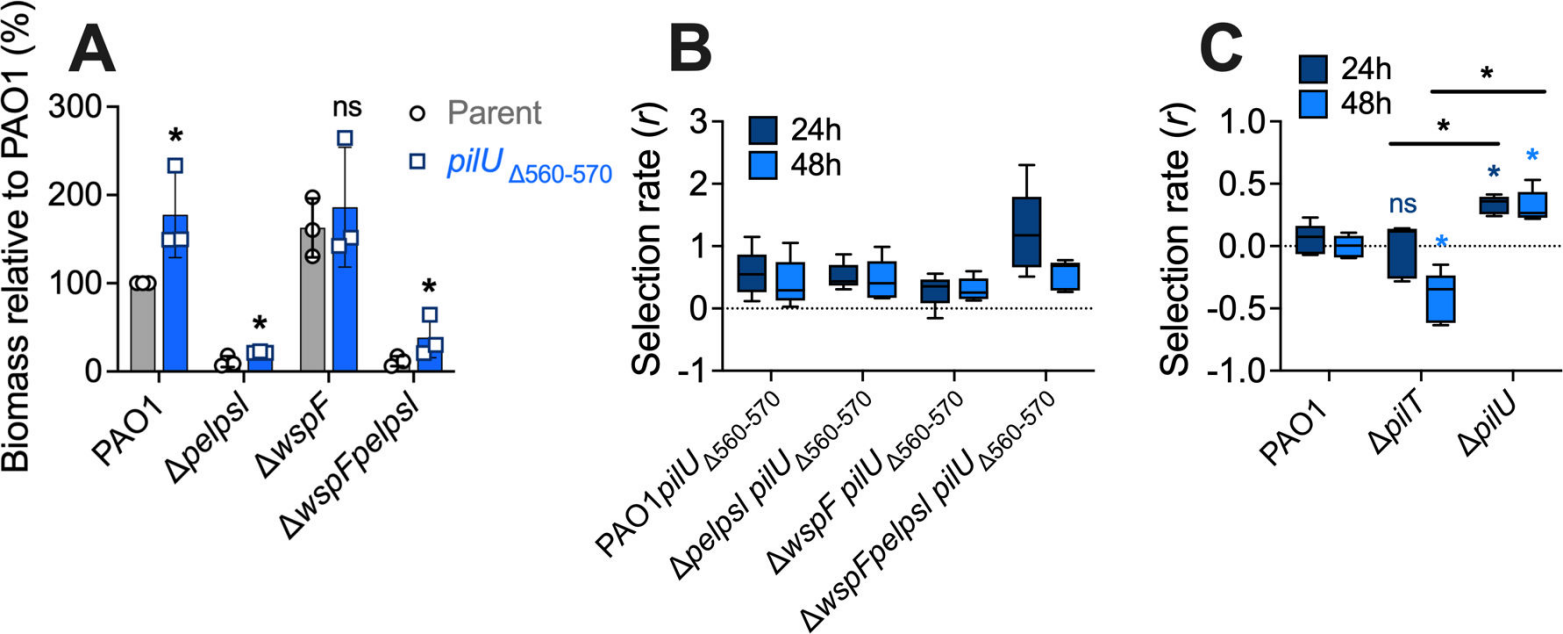
Mutations in *pilU* promote increased biofilm formation and fitness. (**A**) Biofilms were grown in a 96-well plate for 24 h in Jensen’s media. Biofilm biomass was quantified by crystal violet staining. Biomass expressed as a percentage relative to PAO1. Data presented as mean ± SD. Individual data points indicate biological replicates, which are the average of four technical replicates. *N* = 3. Significance was determined using two-tailed unpaired *t*-test, between the parent and mutant. *P-value < 0.05, ns indicates no significance, compared with the parent background. Selection rate (*r*) of (**B**) *pilU*_Δ560-570_ mutants or (**C**) complete *pilT* and *pilU* gene deletions, competed pairwise against the parent in a biofilm for 24 and 48h (dark and light blue, respectively). *N* = 5. (**C**) Significance determined using a two-way ANOVA with a Tukey’s multiple comparison post-hoc test. *P-value < 0.05, ns indicates no significance. Colored *, comparison to PAO1 competition at either 24 or 48 h. Black *, comparison indicated on the graph.

We initially hypothesized that *P. aeruginosa* adaptation to the wound is targeted toward mutations leading to hyperbiofilm phenotypes. To therefore determine if the *pilU*_Δ560-570_ mutation was associated with changes to biofilm formation, biofilms were grown for 24 h in a 96-well plate, and biofilm biomass quantified by crystal violet. In all backgrounds, except for Δ*wspF*, the *pilU*_Δ560-570_ mutation was associated with a significant increase in biofilm formation compared with the parental strain (Fig. 5A). Furthermore, biofilm formation could be restored to that of the parental strain by complementing *pilU in trans* (Fig. S2C), demonstrating that the hyperbiofilm phenotype is due to the loss of PilU. Quantification of biofilm bacteria by colony-forming units revealed that in the Δ*wspFpelpsl* background, the *pilU*_Δ560-570_ mutation did not result in a significant increase in cells in the biofilm (Fig. S4). We therefore predict that the increased biomass is attributed to hyperpiliation of the cells due to a lack of a functional PilU (29), contributing to an EPS with an increased protein component. To determine if the *pilU*_Δ560-570_ mutation was associated with changes in competitive fitness, the mutant was competed against the parent, which was tagged with *lacZ*, in a biofilm bead assay (33). Importantly, the *lacZ* tag did not confer any changes in fitness to the parent strain (Fig. S5). The selection rate of the mutant was determined at 24 and 48 h, according to equation 1. Across both time points and in all backgrounds, the *pilU*_Δ560-570_ mutation was associated with increased competitive fitness, as indicated by *r* > 0.1, compared with the parent (Fig. 5B). Interestingly, the highest fitness of the *pilU*_Δ560-570_ mutation was observed in the Δ*wspFpelpsl* background after 24 h (Fig. 5B).

*P. aeruginosa* encodes a second ATPase, *pilT*, that also powers the retraction of T4P (34). Both *pilT* and *pilU* mutants have a hyperpiliation phenotype (29). To therefore determine if the increased fitness of the *pilU*_Δ560-570_ mutation was due to a general hyperpiliation phenotype, or specific to a *pilU* mutation, complete gene deletions of *pilT* and *pilU* were constructed in PAO1, and competed pairwise against PAO1 tagged with *lacZ* in the biofilm bead assay (33). The selection rate of the mutant was determined at 24 and 48 h, according to equation 1. After 24 h, there was no change in fitness of Δ*pilT*; however, after 48 h, there was a significant decrease in fitness, when compared with the parent competition control (Fig. 5C). In contrast, Δ*pilU* had significantly increased fitness at both 24 and 48h, compared with both the parent control and Δ*pilT* (Fig. 5C), like what was observed for the *pilU*_Δ560-570_ mutation (Fig. 5B). Together, this indicates that the increased fitness associated with the *pilU*_Δ560-570_ mutation is specific to *pilU* and not due to a general hyperpiliation phenotype.

**Fig 6:**
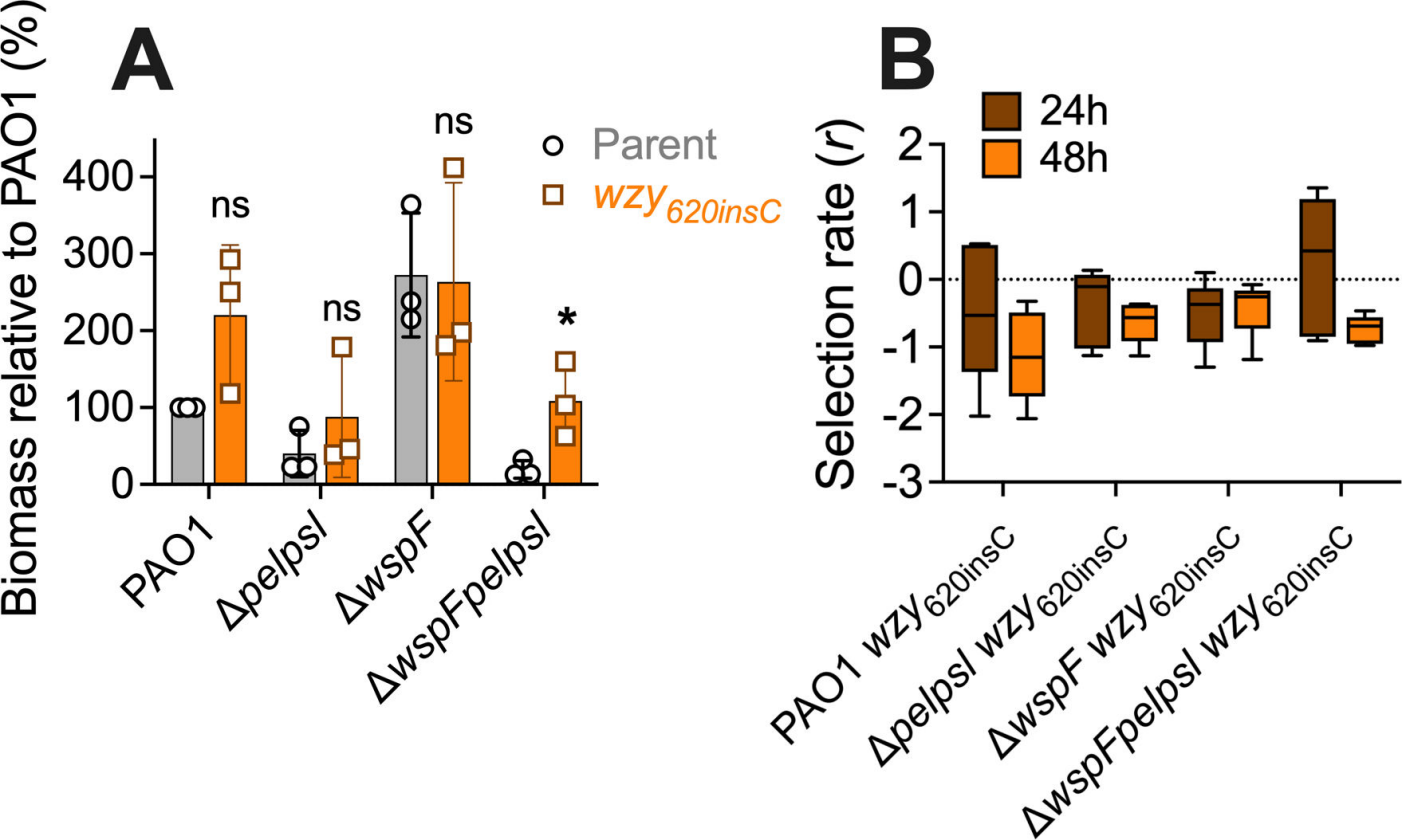
Mutations in *wzy* promote increased biofilm formation but not increased fitness. (**A**) Biofilms were grown in a 96-well plate for 24 h in Jensen’s media. Biofilm biomass was quantified by crystal violet staining. Biomass is expressed as a percentage relative to PAO1. Data presented as mean ± SD. Individual data points indicate biological replicates, which are the average of four technical replicates. *N* = 3. Significance was determined using two-tailed unpaired *t*-test, between the parent and mutant. *P-value < 0.05, ns indicates no significance, compared with the parent background. (**B**) Selection rate (*r*) of *wzy*_620insC_ mutants competed pairwise against the parent in a biofilm for 24 and 48 h (dark and light orange, respectively). *N* = 5.

We next determined phenotypes associated with the *wzy*_620insC_ mutation. To determine if the mutation was associated with changes to biofilm formation, biofilms were grown for 24 h in a 96-well plate, and biofilm biomass quantified by crystal violet. This revealed that in PAO1, Δ*pelpsl* and Δ*wspFpelpsl* backgrounds the *wzy*_620insC_ mutation resulted in an increase in biofilm formation compared with the parental strain (Fig. 6A). However, in PAO1 and Δ*pelpsl*, this was not statistically significant. No difference in biofilm formation was observed for the *wzy*_620insC_ mutation in the Δ*wspF* background (Fig. 6A). Biofilm formation could be restored to that of the parental strain by complementing *wzy in trans* (Fig. S3C), demonstrating that the hyperbiofilm phenotype is due to the loss of Wzy. Enumeration of biofilm bacteria revealed that in the Δ*wspFpelpsl* background, the *wzy*_620insC_ mutant resulted in a significant increase in cells (Fig. S4), accounting for the increased biofilm phenotype in this strain background (Fig. 6A). To determine if the *wzy*_620insC_ mutation was associated with changes in competitive fitness, the mutant was competed against the *lacZ* tagged parent in the biofilm bead assay (33). In all strain backgrounds, at both 24 and 48 h, the *wzy*_620insC_ mutation was associated with decreased fitness, indicating that the mutant was outcompeted by the parent in this assay (Fig. 6B). This is despite the apparent increased biofilm phenotype of *wzy*_620insC_ mutants (Fig. 6A). We predict that a reduced growth rate of *wzy*_620insC_ mutants contributed to the reduced fitness observed when competed against the parental strain (Fig. S6).

**Fig 7:**
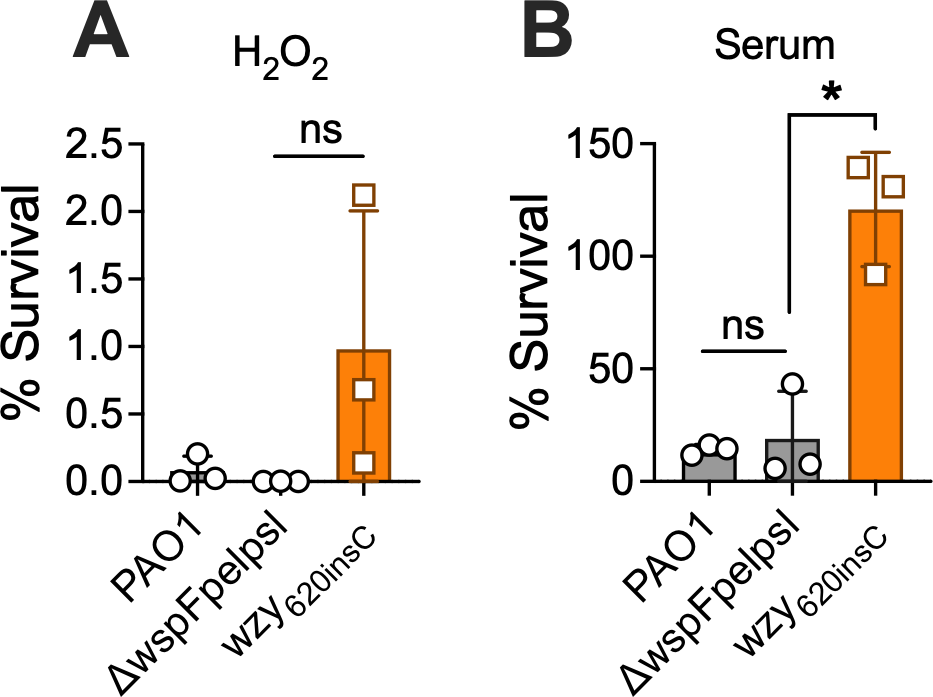
*wzy*_620insC_ mutation is protective against host antimicrobial products. PAO1, Δ*wspFpelpsl*, and Δ*wspFpelpsl wzy*_620insC_ were treated with either (**A**) 25 μM H_2_O_2_ or (**B**) undiluted (100%) serum for 1 h. Bacterial viability was enumerated by CFU/mL and expressed as percent survival relative to the untreated control. *N* = 3. Data presented as mean ± SD. Individual data points indicate biological replicates, which are the average of three technical replicates. *P-value < 0.05, ns indicates no significance. Comparisons are indicated on the graph.

To identify potential fitness advantages of the *wzy*_620insC_ mutation in the wound-like environment, we tested bacterial survival in response to host antimicrobial products. PAO1, Δ*wspFpelpsl,* and Δ*wspFpelpsl wzy*_620insC_ were grown to mid-log and treated with either H_2_O_2_ or serum for 1 h, and bacterial survival was quantified, relative to the untreated control (Fig. 7). PAO1 and Δ*wspFpelpsl* had equivalent levels of survival in both the H_2_O_2_ and serum treatments. However, in the Δ*wspFpelpsl* background, the *wzy*_620insC_ mutation resulted in increased survival to H_2_O_2_ and serum, although this was only statistically significant for serum (Fig. 7). This suggests that the fitness advantage associated with the *wzy*_620insC_ mutation may be due to increased survival to host antimicrobial products, including reactive oxygen species and those present in serum, such as complement. We did not observe any increased survival associated with the *pilU*_Δ560-570_ mutation (Fig. S7).

### Large genomic deletions associated with a subset of SCVs

In addition to the driver SCV mutations described above, we also identified that of the 39 SCVs sequenced, five had acquired unique deletions of large segments (>200 kb) of DNA, with up to 320 consecutive genes deleted (Table 2; Fig. 4). SCVs 54, 57, 58, and 73 had genomic deletions spanning the same region, while SCV 52 had a deletion in the adjacent genomic region (Fig. 4), again highlighting the level of mutational parallelism experienced during infection.

Phenotypic analysis of these SCVs revealed that SCVs 57 and 73 had increased biofilm formation relative to the Δ*wspFpelpsl* parent (Fig. S8A); however, when competed pairwise with Δ*wspFpelpsl* parent in the biofilm bead model, all SCVs with large genome deletions had reduced fitness in this assay (Fig. S8B). Finally, we used SCV 52 and SCV 57 as representative SCVs with large genomic deletions and assessed survival when exposed to host antimicrobials. Neither SCV showed differences in survival when treated with H_2_O_2_ compared with the Δ*wspFpelpsl* parent (Fig. S8C). However, both SCVs showed increased survival when treated with serum (Fig. S8C). In addition to the large genomic deletions, SCV 52 has a base pair insertion in *wzy*, while SCV 57 has a 3,332 base pair deletion, resulting in the deletion of *wbpH*, *wbpG, hisF2, hisH2, wzx*, and *wzy* (Table 2). We therefore hypothesize that the increased tolerance to serum is due to the co-occurring *wzy* mutation, like what we observed for the *wzy*_620insC_ mutation (Fig. 7), rather than due to contributions from the large genomic deletions. Therefore, at this time from our *in vitro* assays, we are unable to determine the fitness benefits associated with these large genomic deletions.

### Evidence for filamentous phage activity

**Fig 8:**
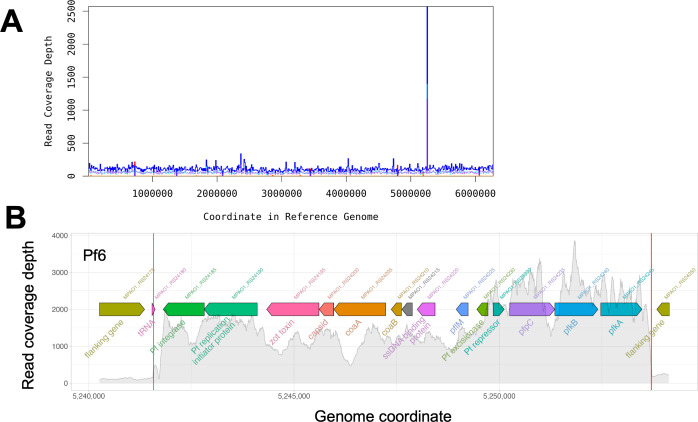
Some SCVs have increased read coverage across Pf6 encoded genes. Read coverage analysis of SCV 50. (**A**) Read coverage of SCV 50 sequences mapped to the reference MPAO1 genome. (**B**) Read coverage of SCV 50 sequences aligning to Pf6 genes in the reference genome. Colored arrows indicate the genes in this region, red lines indicate the genome regions corresponding to the encode prophage. Gray bars indicate the number of sequences aligning to the genome. Similar coverage was observed for SCVs 48, 49, and 50. SCV 69 and 70 had coverage increases in this region but at lower levels. SCV 50 is depicted here as a representative of these isolates.

Finally, we observed that 3 of the 39 sequenced SCVs (SCVs 48, 49, and 50) displayed a significant increase in reads mapping to a specific region of the genome, with approximately 10–20 fold increased coverage (Fig. 8A). Furthermore, SCVs 69 and 70 displayed approximately a fourfold increased coverage at the same genomic region. Review of this region indicated that the reads mapped to genes encoding the Pf6 prophage, which was observed as increased coverage across the entire prophage region, compared with the flanking bacterial genes (Fig. 8B). Notably, we did not observe increased coverage of Pf6 or Pf4 in the Δ*wspFpelpsl* parent strain. (Fig. S9). Together these data suggest that Pf6 filamentous phage was replicating independently of the host bacterial genome. This was not inherent to the parental mutant background, but appeared to be activated during chronic infection of the porcine wound.

## DISCUSSION

Here, we demonstrate that in a porcine full-thickness thermal injury wound model, a triple gene mutant deficient in biofilm formation, Δ*wspFpelpsl,* undergoes adaptive evolution by acquiring mutations that alter the outer membrane; either *pilU*-mediated hyperpiliation or loss of O-antigen, that restores the deficient biofilm phenotype. We predict that these changes to the bacterial outer membrane increase surface adhesion, and binding to neighboring bacterial cells, contributing to the hyperbiofilm phenotype. For *pilU* mutants, this increased biofilm phenotype was associated with enhanced fitness compared with the parent, while for *wzy* mutants this was associated with increased tolerance to host antimicrobial products. It was surprising that the *wzy* mutation conferred increased resistance to serum, as O-antigen-deficient strains of *P. aeruginosa* are typically more sensitive to serum (35). This suggests that there may be a synergistic effect of *wzy* and either *wspF* or *pel* and *psl* mutations that warrant further investigation. To our knowledge, this is the first time these phenotypes and fitness benefits have been identified for *pilU* and *wzy* mutants, contributing to the growing understanding of *P. aeruginosa* adaptation to infection, and the genotype-phenotype associations that are under selection. These data add to the growing evidence that the hyperbiofilm phenotype is adaptive in chronic infections and that *P. aeruginosa* has redundant pathways to generate this phenotype. Our data are also supportive of using variant colony morphology as a screen to identify these adaptive mutations from complex clinical samples (36).

We also observe a striking degree of mutational parallelism (Fig. 4), at both the biosynthetic pathway (T4P and LPS; Fig. 2) and gene (*pilU* and *wzy*; Fig. 3) level, indicating the strong selective pressures experienced by these pathways in a chronic wound infection. We hypothesize that this observed parallelism is the result of independent mutation events because (i) each animal was inoculated with a separate bacterial culture, (ii) wounds were only sampled at a single time point, that is a separate wound was sampled at each time point, and (iii) biopsies were sampled at distinct geographical locations across the 2″ x 2″ wound. However, we cannot rule out that some of the identified SCVs are the result of expansion of mutants that were present in the initial inoculum at low frequency. Regardless, if mutations were present at low frequency in the inoculum, rising to measurable frequency and survival in the wounds still indicates strong selection for these mechanisms. Interestingly, similar mutational parallelism in *wzy* was identified in PAO1 in response to lytic phage predation (37). Previous studies identified mutations in other LPS biosynthetic genes and in T4P genes, also similar to what we observed here (37). Together these data suggest that in our model, mutations in T4P and LPS genes have pleiotropic phenotypes that are selected to expand the niche colonization and survival of *P. aeruginosa*.

Mutations in these pathways are also under selection in *P. aeruginosa* CF lung infections, where similar parallel evolution is observed (2, 3). However, interestingly this mutational parallelism is observed across more extensive genes, with previous studies identifying 52 genes that are repeatedly targeted during CF lung infections (3). By contrast, using the porcine wound model we identified mutational parallelism in four genes, *wspA* (24), *retS* (25), and here in *pilU* and *wzy*. This could be due to different selective pressures between a wound and lung infection environment, differences between adaptive evolution of laboratory strains compared with clinical isolates, or specific to our wound model. However, a caveat of our findings is that we were biased to those mutations that resulted in a variant colony morphology. Utilizing unbiased whole population metagenomic sequencing could reveal more complex evolutionary dynamics in the porcine wound model and will be the focus of future studies.

We also observed significant genome rearrangement of a subpopulation of SCVs during infection. First, we observed large genome deletions, that occurred in similar regions of the genome (Table 2, Fig. 4). Currently, we have been unable to determine the molecular mechanism for these deletions. However, this region of the *P. aeruginosa* genome has been identified as an extended nonessential region, which has been targeted for genome minimization and streamlining (38). Genome reduction of *P. aeruginosa* has been identified in CF lung infections as the isolates become host restricted (39). However, this was observed through the acquisition of pseudogenes, rather than genome deletions as we observed here. Furthermore, host restriction is typically observed over extended time frames of host colonization, with Armbruster et al. observing pseudogene evolution over a 30-month period (39). This suggests that the large genome deletions we observe here may not be a consequence of host restriction. Rather, it seems to be reminiscent of genomic “black holes” that were first described in *Shigella* spp. (40). A large 190-kb genome deletion was identified in *S. flexneria* and enteroinvasive *Escherichia coli* (EIEC) that was absent in non-pathogenic *E. coli*. This large deletion resulted in the removal of the *cadA* locus, conferring increased virulence of *S. flexneria* and EIEC strains (40). Genomic black holes have also been implicated in the evolution of pathogenic *Bacillus*, *Burkholderia, Bordetella, Rickettsiae, Mycobacterium*, and *Chlamydia* spp. by removing anti-virulence genes (41–43). Similarly, large genomic deletions in *P. aeruginosa* have been identified as a response to phage infection, where the deletion removes a gene, or gene cluster, essential for phage entry into the cell, resulting in phage resistance (44, 45). It is therefore interesting to speculate that the large genome deletions that we identified here may similarly confer increased persistence in the wound of the Δ*wspFpelpsl* parent. However, interestingly, large genomic deletions in response to phage infection, in the similar genomic region to what we identified here, have been associated with reduced virulence (46), indicating that the role of these large deletions to bacterial persistence during infection warrants further investigation.

Second, we observed evidence of Pf6 transitioning to the replicative form (Fig. 8). Filamentous phage exist in the host as either two forms; a ssDNA infectious form that is integrated in the host genome as a prophage, or a dsDNA replicative form (RF) that is plasmid-like and replicates independently of the host genome (47). The transition between these forms is associated with changes in the environmental or growth conditions of the host bacterium (48, 49). This suggests that the wound environment induces the RF of Pf6 in Δ*wspFpelpsl*. Furthermore, our detection of the Pf6 RF from the wound is likely an under-representation, as this form can transition back to the prophage form within the host. The increased replication of Pf6 in Δ*wspFpelpsl* SCVs isolated from porcine wounds is notable, as filamentous phage produce phenotypes associated with increased biofilm formation, antibiotic resistance, resistance to phagocytosis and altered mammalian inflammatory responses (47, 50–52). Further, as T4P is the receptor for Pf phage, it is plausible that the T4P mutations observed here may have been selected as defenses against Pf superinfection, which is costly for fitness (53). Finally, filamentous phage, specifically Pf4, has been associated with the SCV phenotype (52, 54). Interestingly, we identified a number of SCVs with mutations in MPAO1_RS28990, which is implicated as a lambda repressor gene (Table 2). Genes with similar annotated domains control lysogeny (55). Together this suggests that Pf6 may contribute to the SCV phenotype in the absence of LPS or T4P mutations.

Interestingly, when identifying the SCV subpopulation from homogenized biopsy tissue we saw substantial variation in the number of SCVs isolated, both across an individual wound and animal (Fig. 1B). This suggests that the microenvironment across the wound is heterogenous, setting up a heterogenous fitness landscape for selection to act upon. This is supported by the heterogenous distribution of microorganisms observed in clinical human wound infections, which is often a complicating factor for diagnosis (56, 57). Additionally, SCVs remained at low frequencies (Fig. 1B), despite having a strong selection rate (Table 1). This could indicate negative frequency-dependent selection, where mutants are more fit when rare (58, 59). This phenomenon has been previously described for *P. aeruginosa* mutants *in vivo* (24), and for *Pseudomonas fluorescens* (60–62) and *Burkholderia cenocepacia* (63) mutants evolved from *in vitro* biofilm experiments. A similar phenomenon has been described as an “insurance hypothesis” by maintaining standing genetic diversity that selection can then act upon to ensure survival and persistence (15). Therefore, rare variants may be an important adaptive strategy of bacteria to ensure survival across fluctuating environments, such as chronic infections.

## MATERIALS AND METHODS

### Bacterial strains and plasmids

Bacterial strains and plasmids used in this study are detailed in Table S1. Mutant and deletion constructs were made using NEBuilder HiFi DNA assembly (NEB). Complementing constructs were made by ligation using T4 ligase (NEB) according to standard protocols. Mutant and deletion constructs were incorporated into the *P. aeruginosa* genome by two-step allelic recombination. Complementation constructs were introduced into *P. aeruginosa* by electroporation. Plasmid selection was maintained using 100 μg/mL ampicillin or 10 μg/mL gentamicin for *E. coli* and 300 μg/mL carbenicillin or 50 μg/mL gentamicin for *P. aeruginosa*.

### Porcine thermal-injury chronic wound model

Protocols were performed in accordance with OSU IACUC approval. Pigs were wounded and monitored, as previously described (27). Briefly, two pigs were subjected to thermal injury by applying a 2 × 2 inch metal dice heated to 150°C for 25 s to the back of the pig to achieve six (three down each side) full-thickness thermal wounds. Three days post-wounding, wounds were inoculated topically with 250 μL 10^8^ CFU/mL of MPAO1Δ*wspF*Δ*pelA*Δ*pslBCD* (12). Three 8-mm punch biopsies were sampled from two wounds from each pig at 7, 14, and 35 days post-inoculation. Biopsies were homogenized in 1 mL PBS and serially diluted to enumerate CFU/g tissue on *Pseudomonas* isolation agar (PIA). From these plates, colonies were also visually screened for variant colony morphologies that deviated from the parental PAO1Δ*wspF*Δ*pelA*Δ*pslBCD* colony phenotype. Using a sterile toothpick, colonies with a variant morphology were selected and passaged twice on Luria agar (LA), and once on PIA to confirm the variant morphology was stable. Confirmed variants were stored at −80°C.

To image the variant colony morphology, 1 μL of an overnight culture of representative variants was spotted onto PIA and incubated overnight at 37°C. Colonies were imaged using a Lecia x stereoscope, fitted with x camera. Images were captured using x software and processed using FIJI (64).

To determine the fitness of the small-colony variants in the wound, the selection rate (*r*) was calculated according to equation 1 (65).


(1) $$r=\frac{\left(ln\frac{M_{x}}{M_{0}})-(ln\frac{A_{x}}{A_{0}}\right)}{T_{x}}$$

where *T* is time at day *x*, and *M* and *A* are the number of mutant and ancestor cells, respectively, at days 0 and *x*. As the number of mutants in the wound at day 0 is unknown, *s* was estimated using mutant-to-parent ratios of 1:10^5^, 1:10^6^, 1:10^7^, and 1:10^8^.

### Sequencing and analysis

DNA extraction and genome sequencing were performed, as previously described (24). Briefly, genomic DNA was extracted using the DNeasy Blood and Tissue kit (Qiagen) following the manufacturer’s protocol. Isolated DNA was sequenced with the library preparation method according to Baym et al. (66) on an Illumina NextSeq 500. Paired-end 2 × 151 sequencing reads were quality filtered and trimmed with Trimmomatic v0.36 (settings: LEADING:20, TRAILING:20, SLIDINGWINDOW:4:20, MINLEN:70) (67), then variants were called with *breseq* v0.30.0 using default settings (68). We used GCF_016107485.1 as the MPAO1 reference assembly.

For Pf6 analysis, raw reads were trimmed using Trimmomatic v0.36 (settings: LEADING:3, TRAILING:3, SLIDINGWINDOW:4:15, MINLEN:36) (67), and reads were mapped using breseq v0.39.0 with default settings (68). We used MPAO1 (GCF_016107485.1) as the reference genome. Read depth was calculated by taking the bam file generated by breseq, and then averaging reads mapped across a 10-bp window using bedtools v2.26.0 (69) and samtools v1.21 (70).

### T4P quantification

Surface-expressed T4P was purified, as previously described (71). Briefly, bacterial strains were grown as a lawn on LB without salt, solidified with 1.5% agar, for 24 h at 37°C. The bacterial lawn was resuspended in PBS and normalized to an OD_600nm_ of 1 in a total volume of 1 mL. The normalized culture was vortexed for 10 min and centrifuged for 20 min at 13,000 rpm. The supernatant was transferred to a new tube and incubated with 150 μL 1 M MgCl_2_ overnight at 4°C. The precipitated T4P was pelleted by centrifugation at 13,000 rpm for 20 min and resuspended in 150 μL PBS supplemented with 1 mM DTT. T4P was isolated from three biological replicates.

To quantify surface expressed T4P, 2 μL of the pilin preparation was spotted onto a nitrocellulose membrane, and allowed to air dry. The membrane was incubated with 5% skim milk in PBS-T for 1 h. T4P were labeled using a α-PilA (72) diluted 1:250 in 5% skim milk, and 0.1 μg/mL goat α-rabbit secondary antibody conjugated to horseradish peroxidase (ThermoFisher Scientific). Blots were detected using SuperSignal West Pico PLUS Chemiluminescent Substrate (ThermoFisher Scientific) according to the manufacturer’s instructions. Blots were visualized by chemiluminescence using a ChemiDoc Imaging System (BioRad). Blots were quantified by densitometry using FIJI (64). Each biological replicate was performed in duplicate.

### LPS western blot

Overnight cultures were normalized to an OD_600nm_ 0.5 in LB and centrifuged at 10,600 × *g* for 10 min. Pellets were resuspended in 1× Laemmli buffer supplemented with β-mercaptoethanol, boiled at 100°C for 15 min, and then allowed to cool to room temperature for 15 min. Proteinase K (10 mg/mL) was added, and samples were incubated at 59°C for 1 h. Samples were frozen at −20°C until further use.

Samples were electrophoresed using a 12% Mini-PROTEAN TGX gel (Biorad) for 1.25 h at 120V. After electrophoresis, samples were transferred to a nitrocellulose membrane and the membrane was blocked for 1 h in 5% nonfat milk at room temperature. Membranes were incubated in mouse anti-PA antigen O5 (My BioSource) overnight at 4°C. After primary antibody incubation, membranes were washed three times and incubated in goat anti-mouse secondary antibody conjugated to HRP for 1 h at room temperature. Membranes were washed three times before chemiluminescent detection with Supersignal Pico (ThermoFisher) was completed. Images were acquired on a ChemiDoc Imaging System (BioRad).

### Biofilm quantification

Overnight cultures were normalized to an OD_600nm_ 0.5 in Jensen’s defined media (pH 7.3) (73). Then, 100 μL was transferred to a well of a 96-well plate and incubated for 24 h at 37°C in a humidified chamber. Biofilms were stained with 120 μL 0.1% crystal violent for 30 min. Biofilms were washed three times in PBS and bound crystal violet dissolved in 150 μL 100% ethanol for 30 min. OD_590nm_ was measured on a SpectraMax i3 plate reader (Molecular Devices). Three biological replicates were performed, each with three technical replicates. Biofilm biomass was expressed as a percentage of PAO1, which was set to 100%.

### Biofilm competition

Parental strains were tagged with *lacZ* on the *attB* site using miniCTX::*lacZ* (74). The vector backbone was excised using pFLP2 (75).

Overnight cultures were normalized to an OD_600nm_ 1, and 50 μL of competing strains was transferred to 5 mL LB containing a 7-mm polystyrene bead and incubated at 37°C in a rolling culture drum. After 24-h incubation, biofilm-coated bead was transferred to 5 mL LB with a second bead and incubated for another 24 h (33). CFUs were enumerated at time 0 (inoculum culture) 24 and 48 h on LA supplemented with 100 μg/mL X-Gal. To quantify CFUs of biofilms, beads were sonicated in 1 mL PBS in a water bath sonicator for 1 min at 50% power. Biofilms were further disrupted by passing the sonicated culture through a 22″ gage needle. The selection coefficient (*s*) was calculated according to equation 1. Five biological replicates were performed, each with three technical replicates.

### Antimicrobial susceptibility

Overnight cultures were normalized to an OD_600nm_ 0.5. Normalized cultures were centrifuged, and the cell pellet resuspended in equal volume of either undiluted, that is 100% pig serum (Giboc) or 25 μM hydrogen peroxide diluted in PBS for 1 h at 37°C. Heat-inactivated serum or PBS treatment was used as a control. Serum was heat inactivated by incubating at 90°C for 10 min. Technical triplicates were serially diluted and enumerated for CFU/mL and expressed as percent survival relative to the appropriate control. Three biological replicates were performed.

### Statistical analysis

Statistical analysis was performed using a one-way analysis of variance (ANOVA) with a Tukey’s multiple comparison post-hoc test, unless otherwise indicated in the figure legend. Analyses were performed using GraphPad Prism v.10 (GraphPad Software). Statistical significance was determined using a *P*-value < 0.05.

## Data Availability

All sequencing data are available in NCBI SRA under BioProject number PRJNA1283160 and BioSample accession numbers SAMN49683420–SAMN49683459.

## SUPPLEMENTAL MATERIAL

Supplemental figures and table jb.00520-25-s0001.docx Figures S1 to S9 and Table S1.

ASM does not own the copyrights to Supplemental Material that may be linked to, or accessed through, an article. The authors have granted ASM a non-exclusive, world-wide license to publish the Supplemental Material files. Please contact the corresponding author directly for reuse.

## References

[1] Kerr KG, Snelling AM. 2009. Pseudomonas aeruginosa: a formidable and ever-present adversary. J Hosp Infect 73:338–344. doi:10.1016/j.jhin.2009.04.020 19699552

[2] Smith EE, Buckley DG, Wu Z, Saenphimmachak C, Hoffman LR, D’Argenio DA, Miller SI, Ramsey BW, Speert DP, Moskowitz SM, Burns JL, Kaul R, Olson MV. 2006. Genetic adaptation by Pseudomonas aeruginosa to the airways of cystic fibrosis patients. Proc Natl Acad Sci USA 103:8487–8492. doi:10.1073/pnas.0602138103 16687478 PMC1482519

[3] Marvig RL, Sommer LM, Molin S, Johansen HK. 2015. Convergent evolution and adaptation of Pseudomonas aeruginosa within patients with cystic fibrosis. Nat Genet 47:57–64. doi:10.1038/ng.3148 25401299

[4] Sousa AM, Pereira MO. 2014. Pseudomonas aeruginosa diversification during infection development in cystic fibrosis lungs-a review. Pathogens 3:680–703. doi:10.3390/pathogens3030680 25438018 PMC4243435

[5] Randall TE, Eckartt K, Kakumanu S, Price-Whelan A, Dietrich LEP, Harrison JJ. 2022. Sensory perception in bacterial cyclic diguanylate signal transduction. J Bacteriol 204:e0043321. doi:10.1128/JB.00433-21 34606374 PMC8846402

[6] Hickman JW, Tifrea DF, Harwood CS. 2005. A chemosensory system that regulates biofilm formation through modulation of cyclic diguanylate levels. Proc Natl Acad Sci USA 102:14422–14427. doi:10.1073/pnas.0507170102 16186483 PMC1234902

[7] Borlee BR, Goldman AD, Murakami K, Samudrala R, Wozniak DJ, Parsek MR. 2010. Pseudomonas aeruginosa uses a cyclic‐di‐GMP‐regulated adhesin to reinforce the biofilm extracellular matrix. Mol Microbiol 75:827–842. doi:10.1111/j.1365-2958.2009.06991.x 20088866 PMC2847200

[8] Ueda A, Wood TK. 2009. Connecting quorum sensing, c-di-GMP, pel polysaccharide, and biofilm formation in Pseudomonas aeruginosa through tyrosine phosphatase TpbA (PA3885). PLoS Pathog 5:e1000483. doi:10.1371/journal.ppat.1000483 19543378 PMC2691606

[9] Malone JG, Jaeger T, Manfredi P, Dötsch A, Blanka A, Bos R, Cornelis GR, Häussler S, Jenal U. 2012. The YfiBNR signal transduction mechanism reveals novel targets for the evolution of persistent Pseudomonas aeruginosa in cystic fibrosis airways. PLoS Pathog 8:e1002760. doi:10.1371/journal.ppat.1002760 22719254 PMC3375315

[10] Jones CJ, Newsom D, Kelly B, Irie Y, Jennings LK, Xu B, Limoli DH, Harrison JJ, Parsek MR, White P, Wozniak DJ. 2014. ChIP-Seq and RNA-seq reveal an amrz-mediated mechanism for cyclic di-GMP synthesis and biofilm development by Pseudomonas aeruginosa. PLoS Pathog 10:e1003984. doi:10.1371/journal.ppat.1003984 24603766 PMC3946381

[11] Moscoso JA, Mikkelsen H, Heeb S, Williams P, Filloux A. 2011. The Pseudomonas aeruginosa sensor RetS switches type III and type VI secretion via c-di-GMP signalling. Environ Microbiol 13:3128–3138. doi:10.1111/j.1462-2920.2011.02595.x 21955777

[12] Harrison JJ, Almblad H, Irie Y, Wolter DJ, Eggleston HC, Randall TE, Kitzman JO, Stackhouse B, Emerson JC, Mcnamara S, Larsen TJ, Shendure J, Hoffman LR, Wozniak DJ, Parsek MR. 2020. Elevated exopolysaccharide levels in Pseudomonas aeruginosa flagellar mutants have implications for biofilm growth and chronic infections. PLoS Genet 16:e1008848. doi:10.1371/journal.pgen.1008848 32530919 PMC7314104

[13] Kirisits MJ, Prost L, Starkey M, Parsek MR. 2005. Characterization of colony morphology variants isolated from Pseudomonas aeruginosa biofilms. Appl Environ Microbiol 71:4809–4821. doi:10.1128/AEM.71.8.4809-4821.2005 16085879 PMC1183349

[14] Drenkard E, Ausubel FM. 2002. Pseudomonas biofilm formation and antibiotic resistance are linked to phenotypic variation. Nature 416:740–743. doi:10.1038/416740a 11961556

[15] Boles BR, Thoendel M, Singh PK. 2004. Self-generated diversity produces “insurance effects” in biofilm communities. Proc Natl Acad Sci USA 101:16630–16635. doi:10.1073/pnas.0407460101 15546998 PMC528905

[16] O’Neal L, Baraquet C, Suo Z, Dreifus JE, Peng Y, Raivio TL, Wozniak DJ, Harwood CS, Parsek MR. 2022. The Wsp system of Pseudomonas aeruginosa links surface sensing and cell envelope stress. Proc Natl Acad Sci USA 119:e2117633119. doi:10.1073/pnas.2117633119 35476526 PMC9170161

[17] Reinhardt A, Köhler T, Wood P, Rohner P, Dumas J-L, Ricou B, van Delden C. 2007. Development and persistence of antimicrobial resistance in Pseudomonas aeruginosa: a longitudinal observation in mechanically ventilated patients. Antimicrob Agents Chemother 51:1341–1350. doi:10.1128/AAC.01278-06 17261619 PMC1855521

[18] Ikeno T, Fukuda K, Ogawa M, Honda M, Tanabe T, Taniguchi H. 2007. Small and rough colony Pseudomonas aeruginosa with elevated biofilm formation ability isolated in hospitalized patients. Microbiol Immunol 51:929–938. doi:10.1111/j.1348-0421.2007.tb03989.x 17951982

[19] Sheehan DJ, Janda JM, Bottone EJ. 1982. Pseudomonas aeruginosa: changes in antibiotic susceptibility, enzymatic activity, and antigenicity among colonial morphotypes. J Clin Microbiol 15:926–930. doi:10.1128/jcm.15.5.926-930.1982 6808021 PMC272215

[20] Tielen P, Wibberg D, Blom J, Rosin N, Meyer A-K, Bunk B, Schobert M, Tüpker R, Schatschneider S, Rückert C, Albersmeier A, Goesmann A, Vorhölter F-J, Jahn D, Pühler A. 2014. Genome sequence of the small-colony variant Pseudomonas aeruginosa MH27, isolated from a chronic urethral catheter infection. Genome Announc. doi:10.1128/genomeA.00161-14 PMC3900893 24459261

[21] Tielen P, Narten M, Rosin N, Biegler I, Haddad I, Hogardt M, Neubauer R, Schobert M, Wiehlmann L, Jahn D. 2011. Genotypic and phenotypic characterization of Pseudomonas aeruginosa isolates from urinary tract infections. Int J Med Microbiol 301:282–292. doi:10.1016/j.ijmm.2010.10.005 21193347

[22] Brock MT, Fedderly GC, Borlee GI, Russell MM, Filipowska LK, Hyatt DR, Ferris RA, Borlee BR. 2017. Pseudomonas aeruginosa variants obtained from veterinary clinical samples reveal a role for cyclic di-GMP in biofilm formation and colony morphology. Microbiology (Reading, Engl) 163:1613–1625. doi:10.1099/mic.0.000541 29034850

[23] Kasatvo AV, Gorovits ES, Kuznetsova MV, Timasheva OA, Sukhanov SG. 2015. Evaluation of biological properties of Pseudomonas aeruginosa strains isolated from patients with sternum and rib osteomyelitis. Zh Mikrobiol Epidemiol Immunobiol 2:69–74. 26016348

[24] Gloag ES, Marshall CW, Snyder D, Lewin GR, Harris JS, Santos-Lopez A, Chaney SB, Whiteley M, Cooper VS, Wozniak DJ. 2019. Pseudomonas aeruginosa interstrain dynamics and selection of hyperbiofilm mutants during a chronic infection. mBio 10:e01698-19. doi:10.1128/mBio.01698-19 31409682 PMC6692513

[25] Marshall CW, Gloag ES, Lim C, Wozniak DJ, Cooper VS. 2021. Rampant prophage movement among transient competitors drives rapid adaptation during infection. Sci Adv 7:eabh1489. doi:10.1126/sciadv.abh1489 34272240 PMC8284892

[26] Colvin KM, Irie Y, Tart CS, Urbano R, Whitney JC, Ryder C, Howell PL, Wozniak DJ, Parsek MR. 2012. The Pel and Psl polysaccharides provide Pseudomonas aeruginosa structural redundancy within the biofilm matrix. Environ Microbiol 14:1913–1928. doi:10.1111/j.1462-2920.2011.02657.x 22176658 PMC3840794

[27] Roy S, Elgharably H, Sinha M, Ganesh K, Chaney S, Mann E, Miller C, Khanna S, Bergdall VK, Powell HM, Cook CH, Gordillo GM, Wozniak DJ, Sen CK. 2014. Mixed-species biofilm compromises wound healing by disrupting epidermal barrier function. J Pathol 233:331–343. doi:10.1002/path.4360 24771509 PMC4380277

[28] Pestrak MJ, Chaney SB, Eggleston HC, Dellos-Nolan S, Dixit S, Mathew-Steiner SS, Roy S, Parsek MR, Sen CK, Wozniak DJ. 2018. Pseudomonas aeruginosa rugose small-colony variants evade host clearance, are hyper-inflammatory, and persist in multiple host environments. PLoS Pathog 14:e1006842. doi:10.1371/journal.ppat.1006842 29394295 PMC5812653

[29] Whitchurch CB, Mattick JS. 1994. Characterization of a gene, pilU, required for twitching motility but not phage sensitivity in Pseudomonas aeruginosa. Mol Microbiol 13:1079–1091. doi:10.1111/j.1365-2958.1994.tb00499.x 7854122

[30] Islam ST, Taylor VL, Qi M, Lam JS. 2010. Membrane topology mapping of the O-antigen flippase (Wzx), polymerase (Wzy), and ligase (WaaL) from Pseudomonas aeruginosa PAO1 reveals novel domain architectures. mBio 1:e00189-10. doi:10.1128/mBio.00189-10 20824106 PMC2932511

[31] Han X, Kennan RM, Davies JK, Reddacliff LA, Dhungyel OP, Whittington RJ, Turnbull L, Whitchurch CB, Rood JI. 2008. Twitching motility is essential for virulence in Dichelobacter nodosus. J Bacteriol 190:3323–3335. doi:10.1128/JB.01807-07 18310333 PMC2347375

[32] Chiang P, Habash M, Burrows LL. 2005. Disparate subcellular localization patterns of Pseudomonas aeruginosa type IV pilus ATPases involved in twitching motility. J Bacteriol 187:829–839. doi:10.1128/JB.187.3.829-839.2005 15659660 PMC545728

[33] Poltak SR, Cooper VS. 2011. Ecological succession in long-term experimentally evolved biofilms produces synergistic communities. ISME J 5:369–378. doi:10.1038/ismej.2010.136 20811470 PMC3105725

[34] Whitchurch CB, Hobbs M, Livingston SP, Krishnapillai V, Mattick JS. 1991. Characterisation of a Pseudomonas aeruginosa twitching motility gene and evidence for a specialised protein export system widespread in eubacteria. Gene 101:33–44. doi:10.1016/0378-1119(91)90221-v 1676385

[35] Huszczynski SM, Lam JS, Khursigara CM. 2019. The role of Pseudomonas aeruginosa lipopolysaccharide in bacterial pathogenesis and physiology. Pathogens 9:6. doi:10.3390/pathogens9010006 31861540 PMC7168646

[36] Branda SS, Vik S, Friedman L, Kolter R. 2005. Biofilms: the matrix revisited. Trends Microbiol 13:20–26. doi:10.1016/j.tim.2004.11.006 15639628

[37] Latino L, Midoux C, Hauck Y, Vergnaud G, Pourcel C. 2016. Pseudolysogeny and sequential mutations build multiresistance to virulent bacteriophages in Pseudomonas aeruginosa. Microbiology (Reading) 162:748–763. doi:10.1099/mic.0.000263 26921273

[38] Csörgő B, León LM, Chau-Ly IJ, Vasquez-Rifo A, Berry JD, Mahendra C, Crawford ED, Lewis JD, Bondy-Denomy J. 2020. A compact Cascade-Cas3 system for targeted genome engineering. Nat Methods 17:1183–1190. doi:10.1038/s41592-020-00980-w 33077967 PMC7611934

[39] Armbruster CR, Marshall CW, Garber AI, Melvin JA, Zemke AC, Moore J, Zamora PF, Li K, Fritz IL, Manko CD, Weaver ML, Gaston JR, Morris A, Methé B, DePas WH, Lee SE, Cooper VS, Bomberger JM. 2021. Adaptation and genomic erosion in fragmented Pseudomonas aeruginosa populations in the sinuses of people with cystic fibrosis. Cell Rep 37:109829. doi:10.1016/j.celrep.2021.109829 34686349 PMC8667756

[40] Maurelli AT, Fernández RE, Bloch CA, Rode CK, Fasano A. 1998. “Black holes” and bacterial pathogenicity: a large genomic deletion that enhances the virulence of Shigella spp. and enteroinvasive Escherichia coli. Proc Natl Acad Sci USA 95:3943–3948. doi:10.1073/pnas.95.7.3943 9520472 PMC19942

[41] Salimiyan RK, Farsiani H. 2022. Black holes”,“genome fluidity”, and evolution of bacterial species. Rev Clin Med 9:146–155. doi:10.22038/rcm.2023.66126.1404

[42] Day WA, Maurelli AT. 2014. Black holes and antivirulence genes: selection for gene loss as part of the evolution of bacterial pathogens, p 109–122. *In* Evolution of microbial pathogens. ASM Press, Washington, DC, USA.

[43] Maurelli AT. 2007. Black holes, antivirulence genes, and gene inactivation in the evolution of bacterial pathogens. FEMS Microbiol Lett 267:1–8. doi:10.1111/j.1574-6968.2006.00526.x 17233672

[44] Latino L, Essoh C, Blouin Y, Vu Thien H, Pourcel C. 2014. A novel Pseudomonas aeruginosa bacteriophage, Ab31, a chimera formed from temperate phage PAJU2 and P. putida lytic phage AF: characteristics and mechanism of bacterial resistance. PLoS One 9:e93777. doi:10.1371/journal.pone.0093777 24699529 PMC3974807

[45] Le S, Yao X, Lu S, Tan Y, Rao X, Li M, Jin X, Wang J, Zhao Y, Wu NC, Lux R, He X, Shi W, Hu F. 2014. Chromosomal DNA deletion confers phage resistance to Pseudomonas aeruginosa. Sci Rep 4:4738. doi:10.1038/srep04738 24770387 PMC4001099

[46] Menon ND, Penziner S, Montaño ET, Zurich R, Pride DT, Nair BG, Kumar GB, Nizet V. 2022. Increased innate immune susceptibility in hyperpigmented bacteriophage-resistant mutants of Pseudomonas aeruginosa. Antimicrob Agents Chemother 66:e0023922. doi:10.1128/aac.00239-22 35862755 PMC9380547

[47] Secor Patrick R, Burgener EB, Kinnersley M, Jennings LK, Roman-Cruz V, Popescu M, Van Belleghem JD, Haddock N, Copeland C, Michaels LA, de Vries CR, Chen Q, Pourtois J, Wheeler TJ, Milla CE, Bollyky PL. 2020. Pf bacteriophage and their impact on Pseudomonas virulence, mammalian immunity, and chronic infections. Front Immunol 11:244. doi:10.3389/fimmu.2020.00244 32153575 PMC7047154

[48] Xue H, Xu Y, Boucher Y, Polz MF. 2012. High frequency of a novel filamentous phage, VCY φ, within an environmental Vibrio cholerae population. Appl Environ Microbiol 78:28–33. doi:10.1128/AEM.06297-11 22020507 PMC3255608

[49] Jian H, Xu J, Xiao X, Wang F. 2012. Dynamic modulation of DNA replication and gene transcription in deep-sea filamentous phage SW1 in response to changes of host growth and temperature. PLoS One 7:e41578. doi:10.1371/journal.pone.0041578 22870232 PMC3411601

[50] Secor P.R, Michaels LA, Smigiel KS, Rohani MG, Jennings LK, Hisert KB, Arrigoni A, Braun KR, Birkland TP, Lai Y, Hallstrand TS, Bollyky PL, Singh PK, Parks WC. 2017. Filamentous bacteriophage produced by Pseudomonas aeruginosa alters the inflammatory response and promotes noninvasive infection in vivo. Infect Immun 85:e00648-16. doi:10.1128/IAI.00648-16 27795361 PMC5203648

[51] Burgener EB, Sweere JM, Bach MS, Secor PR, Haddock N, Jennings LK, Marvig RL, Johansen HK, Rossi E, Cao X, Tian L, Nedelec L, Molin S, Bollyky PL, Milla CE. 2019. Filamentous bacteriophages are associated with chronic Pseudomonas lung infections and antibiotic resistance in cystic fibrosis. Sci Transl Med 11:eaau9748. doi:10.1126/scitranslmed.aau9748 30996083 PMC7021451

[52] Secor PR, Sweere JM, Michaels LA, Malkovskiy AV, Lazzareschi D, Katznelson E, Rajadas J, Birnbaum ME, Arrigoni A, Braun KR, Evanko SP, Stevens DA, Kaminsky W, Singh PK, Parks WC, Bollyky PL. 2015. Filamentous bacteriophage promote biofilm assembly and function. Cell Host Microbe 18:549–559. doi:10.1016/j.chom.2015.10.013 26567508 PMC4653043

[53] Kubota N, Scribner MR, Cooper VS. 2025. Filamentous cheater phages drive bacterial and phage populations to lower fitness. bioRxiv. doi:10.1101/2025.04.01.646652 41043417

[54] Webb JS, Lau M, Kjelleberg S. 2004. Bacteriophage and phenotypic variation in Pseudomonas aeruginosa biofilm development. J Bacteriol 186:8066–8073. doi:10.1128/JB.186.23.8066-8073.2004 15547279 PMC529096

[55] Bell CE, Frescura P, Hochschild A, Lewis M. 2000. Crystal structure of the lambda repressor C-terminal domain provides a model for cooperative operator binding. Cell 101:801–811. doi:10.1016/s0092-8674(00)80891-0 10892750

[56] Jakobsen TH, Xu Y, Bay L, Schønheyder HC, Jakobsen T, Bjarnsholt T, Thomsen TR. 2021. Sampling challenges in diagnosis of chronic bacterial infections. J Med Microbiol 70. doi:10.1099/jmm.0.001302 33410733

[57] Malone M, Bjarnsholt T, McBain AJ, James GA, Stoodley P, Leaper D, Tachi M, Schultz G, Swanson T, Wolcott RD. 2017. The prevalence of biofilms in chronic wounds: a systematic review and meta-analysis of published data. J Wound Care 26:20–25. doi:10.12968/jowc.2017.26.1.20 28103163

[58] Brisson D. 2018. Negative frequency-dependent selection is frequently confounding. Front Ecol Evol 6:10. doi:10.3389/fevo.2018.00010 34395455 PMC8360343

[59] Christie MR, McNickle GG. 2023. Negative frequency dependent selection unites ecology and evolution. Ecol Evol 13:e10327. doi:10.1002/ece3.10327 37484931 PMC10361363

[60] Rainey PB, Rainey K. 2003. Evolution of cooperation and conflict in experimental bacterial populations. Nature 425:72–74. doi:10.1038/nature01906 12955142

[61] Rainey PB, Travisano M. 1998. Adaptive radiation in a heterogeneous environment. Nature 394:69–72. doi:10.1038/27900 9665128

[62] Kim W, Levy SB, Foster KR. 2016. Rapid radiation in bacteria leads to a division of labour. Nat Commun 7:10508. doi:10.1038/ncomms10508 26852925 PMC4748119

[63] Turner CB, Buskirk SW, Harris KB, Cooper VS. 2020. Negative frequency-dependent selection maintains coexisting genotypes during fluctuating selection. Mol Ecol 29:138–148. doi:10.1111/mec.15307 31725941 PMC6952539

[64] Schindelin J, Arganda-Carreras I, Frise E, Kaynig V, Longair M, Pietzsch T, Preibisch S, Rueden C, Saalfeld S, Schmid B, Tinevez J-Y, White DJ, Hartenstein V, Eliceiri K, Tomancak P, Cardona A. 2012. Fiji: an open-source platform for biological-image analysis. Nat Methods 9:676–682. doi:10.1038/nmeth.2019 22743772 PMC3855844

[65] Cooper VS. 2018. Experimental evolution as a high-throughput screen for genetic adaptations. mSphere 3. doi:10.1128/mSphere.00121-18 PMC5956144 29743200

[66] Baym M, Kryazhimskiy S, Lieberman TD, Chung H, Desai MM, Kishony R. 2015. Inexpensive multiplexed library preparation for megabase-sized genomes. PLoS One 10:e0128036. doi:10.1371/journal.pone.0128036 26000737 PMC4441430

[67] Bolger AM, Lohse M, Usadel B. 2014. Trimmomatic: a flexible trimmer for Illumina sequence data. Bioinformatics 30:2114–2120. doi:10.1093/bioinformatics/btu170 24695404 PMC4103590

[68] Deatherage DE, Barrick JE. 2014. Identification of mutations in laboratory-evolved microbes from next-generation sequencing data using breseq. Methods Mol Biol 1151:165–188. doi:10.1007/978-1-4939-0554-6_12 24838886 PMC4239701

[69] Quinlan AR, Hall IM. 2010. BEDTools: a flexible suite of utilities for comparing genomic features. Bioinformatics 26:841–842. doi:10.1093/bioinformatics/btq033 20110278 PMC2832824

[70] Li H, Handsaker B, Wysoker A, Fennell T, Ruan J, Homer N, Marth G, Abecasis G, Durbin R, 1000 Genome Project Data Processing Subgroup. 2009. The sequence alignment/map format and SAMtools. Bioinformatics 25:2078–2079. doi:10.1093/bioinformatics/btp352 19505943 PMC2723002

[71] Whitchurch CB, Leech AJ, Young MD, Kennedy D, Sargent JL, Bertrand JJ, Semmler ABT, Mellick AS, Martin PR, Alm RA, Hobbs M, Beatson SA, Huang B, Nguyen L, Commolli JC, Engel JN, Darzins A, Mattick JS. 2004. Characterization of a complex chemosensory signal transduction system which controls twitching motility in Pseudomonas aeruginosa. Mol Microbiol 52:873–893. doi:10.1111/j.1365-2958.2004.04026.x 15101991

[72] Baynham PJ, Ramsey DM, Gvozdyev BV, Cordonnier EM, Wozniak DJ. 2006. The Pseudomonas aeruginosa ribbon-helix-helix DNA-binding protein AlgZ (AmrZ) controls twitching motility and biogenesis of type IV pili. J Bacteriol 188:132–140. doi:10.1128/JB.188.1.132-140.2006 16352829 PMC1317580

[73] Jensen SE, Fecycz IT, Campbell JN. 1980. Nutritional factors controlling exocellular protease production by Pseudomonas aeruginosa. J Bacteriol 144:844–847. doi:10.1128/jb.144.2.844-847.1980 6776099 PMC294740

[74] Hoang TT, Kutchma AJ, Becher A, Schweizer HP. 2000. Integration-proficient plasmids for Pseudomonas aeruginosa: site-specific integration and use for engineering of reporter and expression strains. Plasmid 43:59–72. doi:10.1006/plas.1999.1441 10610820

[75] Hoang TT, Karkhoff-Schweizer RR, Kutchma AJ, Schweizer HP. 1998. A broad-host-range Flp-FRT recombination system for site-specific excision of chromosomally-located DNA sequences: application for isolation of unmarked Pseudomonas aeruginosa mutants. Gene 212:77–86. doi:10.1016/s0378-1119(98)00130-9 9661666

